# Barriers, Adoption, and Use of a Bike-Sharing System: A Market-Segment Approach to Current and Potential Users in Montréal, Canada

**DOI:** 10.1177/03611981251340377

**Published:** 2025-06-26

**Authors:** Panagiotis Goudis, Rodrigo Victoriano-Habit, Thiago Carvalho, Ahmed El-Geneidy

**Affiliations:** 1 School of Urban Planning, McGill University, Montreal, QC, Canada

**Keywords:** pedestrians, bicycles, human factors, bicycle transportation, bikesharing, electric bicycles, level of service

## Abstract

Bike-sharing systems have gained considerable traction as a solution to many urban transport challenges. (Note that, in this paper, “bike” means “bicycle,” not “motorbike”.) Limited research has explored the market dynamics driving bike-sharing usage among different population segments. This study is the first to apply a market-segmenting approach to analyze factors influencing both existing and potential users’ adoption of a bike-sharing system in Montréal, Canada. Utilizing a bilingual online survey conducted in spring, 2024, this research investigates the factors that limit or prevent the use of a large-scale bicycle-sharing system—Bixi—among different population groups. This work applies factor and k-means cluster analyses to identify distinct profiles within existing users (*N* = 561) and non-users (*N* = 763) of Bixi. The findings reveal key barriers faced both by users and non-users, particularly highlighting the need for expanding station availability in underserved areas. These findings address important equity issues. Measures to address cost and membership-related challenges would have the greatest impact on bringing low-income non-users into the system, while improving mobile technology accessibility would have the most significant effect on increasing adoption among younger, low-income groups. These insights can be of interest to policy developers aiming to expand bike-sharing systems service quality, enhance its resilience, and promote more equitable outcomes.

Bike-sharing systems have gained considerable traction as innovative solutions to urban transport challenges. (Note that, in this paper, “bike” means “bicycle,” not “motorbike”.) They have been found to promote active and sustainable mobility as well as to derive positive health benefits for users (*1*, *2*). Despite bike-sharing systems primarily substituting other active trips, they also have shown potential to replace motorized trips, contributing to more sustainable transportation systems (*3*, *4*). They have acquired a relevant role in urban transport as they provide additional flexibility and convenience for users, enabling the use of bicycles with lower costs and responsibilities compared with personal bicycles (*3*, *5*, *6*). Despite their potential, bike-sharing programs often encounter hurdles that impede their effectiveness and sustainability. Whilst there is a general growth in the number of cities around the world implementing bicycle-sharing programs, many of them face challenges to survive in the long term because of insufficient coverage area, system capacity, inconvenient payment structures, and lack of governmental support (*7*).

Multiple studies have investigated a diversity of factors that affect the effectiveness of these systems in providing an attractive service and capturing ridership (*8* [Bibr bibr9-03611981251340377][Bibr bibr10-03611981251340377][Bibr bibr11-03611981251340377][Bibr bibr12-03611981251340377]–*13*). However, few studies have explored the bike-sharing market to better understand the differing elements that can influence the use of bike-sharing systems for different segments of the population (*14* [Bibr bibr15-03611981251340377][Bibr bibr16-03611981251340377]–*17*). To the authors’ knowledge, this work is the first to use a market-segmenting approach to explicitly analyze the factors that could influence both existing users’ and non-users’ bike-sharing usage or adoption.

Using a survey sample of 1,324 cyclists collected in the spring of 2024 in Montréal, Canada, this study delves into the potential factors limiting cyclists’ usage of a large-scale bike-sharing system. Responses by cyclists are analyzed, as they are more likely to have prior experience with biking, providing valuable insights into the perceived barriers that may affect bike-sharing adoption. It is important to recognize that users’ and non-users’ perceptions may differ because of varying levels of experience with the system. Because of this, users (*N* = 561) and non-users (*N* = 763) of the bicycle sharing system in Montréal are analyzed separately to understand their unique reasons and challenges potentially limiting their use. A combination of factor and k-means cluster analysis is used to derive profiles for each subsample. In the case of current users, these profiles are identified based on the challenges they face when using the system, as well as on potential improvements to the system that would make them use it more. For the non-user subsample, segments are identified based on their reasons for not using the system, as well as potential improvements that would make them adopt it in the future. The findings in this work offer critical insights into the barriers and challenges faced by users and non-users, highlighting targeted areas for improvement. These contributions are crucial for public policymaking, guiding the future implementation of bike-sharing systems, offering actionable strategies to enhance quality and coverage of service, increasing system resilience, and effectively promoting equitable access.

## Literature Review

In recent years, bike-sharing systems have been increasingly analyzed within the context of integrated urban mobility solutions, including frameworks such as Mobility-as-a-Service (*18 [Bibr bibr19-03611981251340377]*–*20*). Much of the literature focuses on the contributions of bike-sharing systems to sustainable urban transport, examining how they complement other transportation modes and contribute to environmental goals (*21*, *22*). More specifically, understanding the factors that influence bike-sharing ridership can help improve system design, increase accessibility, and promote greater adoption among diverse urban populations (*1*, *23*). This section reviews the literature analyzing factors influencing bike-sharing ridership and examines the different market segments that shape their use, focusing on the key dynamics driving adoption and barriers to usage.

### Factors Influencing Bike-Sharing Ridership

The number of bike-sharing systems has been increasing around the world, although many fail to survive in the long term (*7*). In this context, researchers have been investigating factors encouraging ridership to ensure system longevity. Bike-sharing ridership can be influenced by factors both intrinsic and extraneous to the service provided (*24*). Intrinsic factors can include bike and station availability, payment and membership structure, and how the system connects to the transit system (*25* [Bibr bibr26-03611981251340377]–*27*). Extraneous factors can relate to weather conditions and land use and built environment characteristics not controlled by the service provider (*8*, *24*). The factors identified in the literature are used to guide the choice of variables included in the analyses conducted in this paper.

Intrinsic to the system, the distribution of stations—both in relation to each other and to potential users— increases bike-sharing demand (*25*). While more bikes are generally linked to higher ridership, Zhao et al. warn that an oversupply can lead to inefficiencies in the system (*11*). Beyond station and bike availability, geographic coverage and the scale of the system also affect ridership levels positively, as broader coverage and a larger system can attract more users (*28*).

Low density of stations and proximity to bus stops and metro stations can negatively affect user experience and increase system vulnerability if stations/docks become faulty (*29*). A benefit of proximity of bike-sharing to public transit is that they can serve as an alternative in case of transit disruptions enhancing the resilience of the whole transport network (*30*). Moreover, the number of docks within a metro/rail station catchment area has been found to increase bike-sharing ridership (*10*, *30*) and people with a yearly bike-sharing membership are more likely to do trips integrating cycling and transit (*25*).

Concerning payment methods, users prefer convenience. In a study across bike-sharing systems in 106 cities, Zhang et al. highlight that riders prefer systems where the first few hours are free, followed by a fixed rate (*7*). Riders preferred paying by using a smartcard or coins rather than their phone (*7*, *11*). Fare structure, as well as the payment methods accepted, can significantly influence the likelihood of success of a bike-sharing system. However, while the fare structure is more critical during the initial stages of implementation, payment methods become more prominent once the system becomes more stable (*7*).

Extrinsic to this system, weather conditions can strongly affect trip generation rates (*8*). Inclement weather has a detrimental effect on the rates of daily recreational and commuting trips (*31*). On the other hand, warmer temperatures are usually correlated with higher trip counts except when associated with humidity levels higher than 60% (*23*, *32*). As a result, bike-sharing demand changes seasonally, with bikes and stations becoming closed or more idle during snowy and rainy winter periods (*33*).

Continuous cycling paths separated from traffic encourage cycling because of increased comfort and safety (*34* [Bibr bibr35-03611981251340377][Bibr bibr36-03611981251340377]–*37*). Consequently, the availability of cycling infrastructure that promotes safety around stations has been linked to increased ridership and improved public perception of bike-sharing systems (*34*, *38*). Xu and Chow report that the addition of 1 mile of cycling lanes led to an average increase of 102 bike-sharing trips in New York City (*9*). Results were more prominent within Manhattan where infrastructure improvements led to 285 additional trips. Stronger results in Manhattan are likely related to population density and higher density of points of interest, which are linked in the literature to increased ridership levels (*10*, *24*, *39*).

### Segments of the Bike-Sharing Market

Although many cyclist typologies have been introduced in the literature, few studies have explored segments of the bike-sharing market (*14–17*, *40–42*). Studies in this selection mostly segment the market based on travel behavior characteristics, perceptions/intentions toward a specific bike-sharing system, or both. Xing et al. use data from the bike-sharing operator in Shanghai, China, which are linked to points of interest surrounding trip destinations (*14*). K-means clustering is applied to find segments based on patterns of trip purpose, namely dining, transportation (i.e., as part of multi-modal trips), shopping, work, and residential. Orvin and Fatmi examine profiles of dockless bike-sharing users in Kelowna, Canada (*16*). People are grouped based on a latent segmentation-based logit model finding two segments based on socio-demographic characteristics and frequency of use. Their model indicates that, in the region, low-income women riders tend to use the service more frequently than younger high-income men riders. However, this finding is not representative of the literature at large, as most studies indicate that bike-sharing members tend to be male, high-income, and young (*1*, *5*).

In addition to behavioral and sociodemographic characteristics, Morton includes psychographic information in defining bike-sharing segments in London, UK (*15*). Four groups are identified based on perceptions of service attributes, satisfaction levels, and their willingness to reuse and to recommend services. Policy implications are derived focusing on how to improve satisfaction levels. For instance, the group denominated as “low frequenters” is determined to be price-sensitive; therefore, the authors believe that pricing structures reducing cost of access would increase their satisfaction.

Mohiuddin et al. is the only study exploring increasing bike-sharing use (*17*). Their analysis centers around equity-seeking groups in the greater Sacramento region in the U.S. Their findings indicate that low-income and zero-car households tend to use bike-sharing services more frequently and should be the focus of campaigns aiming to increase bike-sharing adoption in the region. Even so, their study focusses only on samples of bike-sharing users while disregarding non-users.

This study uniquely contributes to the literature by analyzing both user and non-user profiles in relation to the factors that limit or facilitate bike-sharing usage and adoption. By employing a market-segmenting approach, this research provides a nuanced understanding of the specific barriers and motivations of these distinct groups. This approach is crucial for identifying strategies to expand service coverage and improve system quality across diverse segments of the population, ultimately fostering greater equity and resilience within bike-sharing systems.

## Case Study

**Figure 1.**
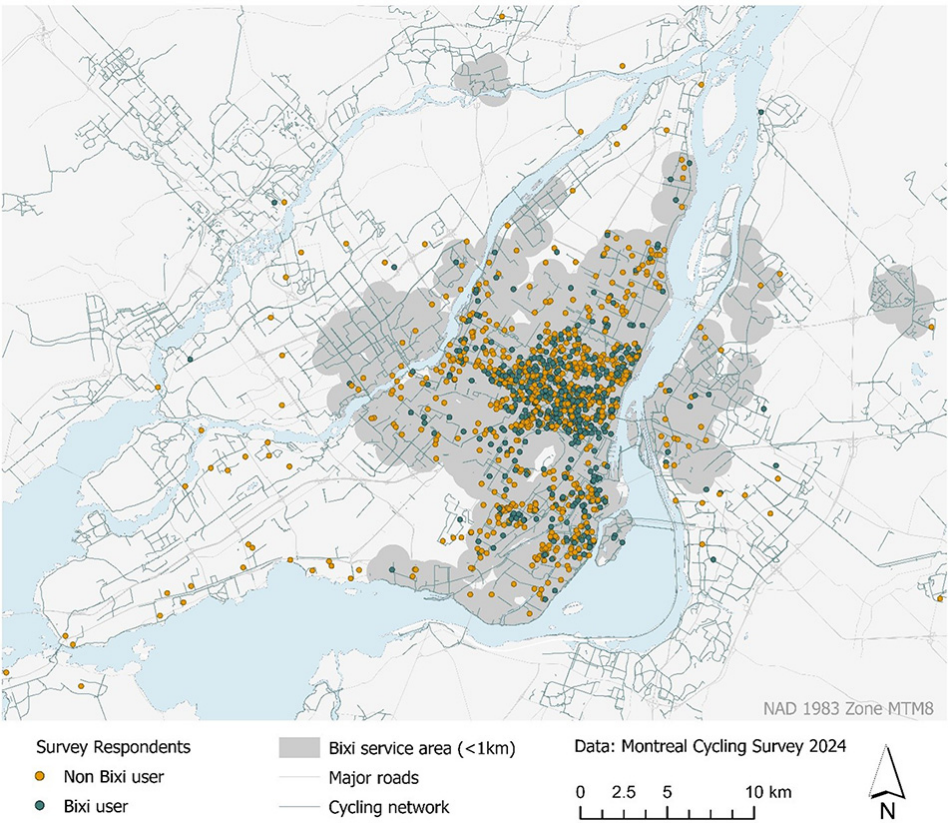
Survey respondents and Bixi system service area.

The bike-sharing system in Montréal, known as Bixi, was launched in 2009 as the first large-scale docked bike-sharing system in North America with 3,000 bicycles and 300 stations. The number of Bixi stations and service area has been growing consistently since its inauguration. Currently, it operates with over 900 stations and more than 11,000 bicycles, including both traditional (*N* = 8,400) and electric models (*N* = 2,600) (*43*). These bicycles, like most of those used in bike-sharing systems, have a robust design that is heavier than most personal bicycles. Bixi covers a significant portion of the core section of the Greater Montréal Area, as shown in Figure 1. The pricing scheme for the Bixi system considers two membership rates and individual trip rates. In 2024, monthly memberships were priced at $22 Canadian dollars (CAD), while a seasonal membership, valid from April to November, is priced at $107 CAD. Both memberships include unlimited trips with a time limit of 45 min per trip. Individual trips incur a base cost of $1.35 CAD plus $ 0.20 CAD per minute of use for a traditional bicycle, and $ 0.35 CAD per minute for an electric bicycle. A $100 CAD hold is placed on the credit card for individual rides as a security deposit (*44*).

Bixi services have been able to attract 14% of the population living within 250 m of a docking station (*45*). In examining Bixi usage flows, Faghih-Imani et al. report that people are more likely to use the service under good weather conditions, leading ridership to peak in the summer months (*23*, *24*). Precipitation, on the other hand, causes a significant reduction in trip generation rates up to 3 h after it has stopped (*23*). Land use and built environment factors also influence Bixi usage. Stations in the vicinity of points of interest (i.e., restaurants, services, and universities) and higher population density correlate with increased Bixi trip rates (*24*). Conversely, ridership decreases on weekends, which is explained by the system being used mostly for utilitarian purposes (*23*, *24*). This behavior pattern is likely a reflection of stations being spatially concentrated and available mostly in central areas (*25*). Consequently, Bixi flows tend to decrease farther from the central business district. In trying to increase Bixi usage flows, previous research indicates that providing additional stations has a stronger impact on usage than increased station capacity (*24*).

## Data

The primary dataset used in this study comes from an online bilingual survey administered in the Greater Montréal Area during spring 2024. An online advertisement campaign on various social media platforms was used to reach cyclists 18 years or older in Montréal. A total of 3,121 emails were sent to cyclists who previously provided their emails in other surveys conducted by the Transportation Research at McGill (TRAM) team and consented to participate in future studies by the team. Incentives to complete the survey were included by giving respondents the possibility of entering a draw with the chance of winning a prize. This multi-faceted recruitment strategy, including online advertisements across various social media platforms, email lists, and incentives, responds to recommendations by Dillman et al. to enhance representativeness and improve survey participation (*46*). This recruitment process resulted in the collection of 1,530 complete responses.

In the Greater Montreal Area, both English and French are prevalent languages. The most commonly spoken language varies significantly across neighborhoods, leading to a correlation with various built-environment and sociodemographic characteristics (*47*, *48*). As a result, the use of a French-English bilingual survey is an essential component of ensuring a representative sample of Montréal. Moreover, the survey was designed and thoroughly reviewed by bilingual members of the TRAM team to ensure consistency between the French and English versions, thereby minimizing potential biases related to wording or interpretation. A thorough data-cleaning process was applied to these responses to ensure the reliability of the final sample. The exclusion criteria included removing multiple responses coming from the same email or IP address, and removing respondents whose home location was outside the Montréal census metropolitan area or in invalid locations such as on bodies of water.

The application of all filters in the data-cleaning process resulted in a validated sample of 1,426 responses. This work focuses only on survey participants who reported having cycled at least once in the last 12 months, resulting in a final sample size of 1,324 valid observations, which is comparable to several other studies within the cycling behavior literature (*15 [Bibr bibr16-03611981251340377]*–*17*, *42*). For the purposes of this work, this sample was subdivided into two groups: Bixi users (*N* = 561) and non-Bixi users (*N* = 763). These two groups were defined based on the question “Have you used Bixi at least once in the last 12 months?”. For both subsamples, the survey included questions about cycling attitudes and behavior. A question was asked about the number of trips performed in the last week by personal bicycle, traditional Bixi, and electric Bixi. The same sociodemographic and home location questions were asked for both subsamples. For the sample of Bixi users, the survey included several questions pertaining to the potential challenges respondents faced in using the bicycle-sharing system, as well as questions about potential improvements to the system that would make them use it more. For the non-Bixi-user sample, the survey asked multiple questions about the reasons for not using Bixi, as well as potential improvements that would make them use it in the future.

Complementary data was used to account for cycling accessibility. The BikeScore index was retrieved from walkscore.com for each respondent’s home location. BikeScore is a popular publicly available measure of cycling accessibility which has repeatedly been used in the cycling literature and has shown reliability in predicting urban cycling patterns (*49*). This index is based on four equally weighted components to measure bikeability: presence of bike lanes, topography and inclination, destinations and network connectivity, and cycling commuting mode share (*50*). The destination and connectivity component is calculated as an adaptation of the WalkScore index, which has also been repeatedly tested in the land-use and transport literature (*51*). This corresponds to a gravity-based measure that considers several types of amenity, including grocery stores, schools, parks, and restaurants. The value of BikeScore ranges from 0 to 100, where higher values indicate higher levels of cycling accessibility.

## Methods

In this study, market segments are identified within two groups: Bixi users (*N* = 561) and non-Bixi users (*N* = 763). The overall goal is to understand factors that would lead already users to increase their usage, and non-users to adopt the service. Analyzing the responses of existing cyclists allows to understand the barriers specifically associated with bike-sharing systems, as opposed to cycling barriers in general. To segment each group, a combination of a factor and k-means cluster analysis is conducted. The combination of factor analysis and k-means clustering has been widely used across many fields in defining market segments. In transportation, some examples include identifying transit markets and sport utility vehicle buying behaviors (*52* [Bibr bibr53-03611981251340377][Bibr bibr54-03611981251340377]–*55*). Additionally, k-means clustering requires less computation power than other techniques.

### Exploratory Factor Analysis

Factor analysis identifies the smallest number of unique underlying latent constructs within the covariance structure of a set of variables (*56*). In this work, this technique is applied to reduce the number of variables to be analyzed with a minimum loss of information. For the group of Bixi users, variables related to challenges experienced by the respondents are included, as well as variables related to perceived areas of service improvement. Areas of service improvement are explored among non-Bixi users along with reasons for not using the service. Variables were selected with the aim of identifying critical service improvement areas among different market segments.

For each group, principal components exploratory factor analysis is conducted using the *psych* and *factoextra* packages in R based on polychoric correlation matrices. This correlation type was chosen because the studied variables were collected in a dichotomous scale (yes/no). Polychoric correlation is found to better deal with variables with less than five categories, as well as to reduce the influence of non-normality on results (*57*). The number of factors extracted was defined based on the latent root criterion (eigenvalues ≥ 1) and the parallel analysis, which has been proven to perform better than scree plots in determining the number of factors to be retained (*58*). Varimax was applied as the rotation method to reduce the likelihood of variables loading highly in multiple factors (*56*). This rotation method assumes factor scores to be uncorrelated, which is supported by our data. The correlation among factor scores is at most 5% for the Bixi sample and 8% for the non-Bixi-user sample.

Variables with loadings lower than 0.5 were removed from the analysis as they lacked significance (*56*). While the literature does not establish a clear-cut threshold for identifying significant factor loadings, with some scholars accepting scores as low as 0.4 as acceptable, we opted for a more conservative 0.5 threshold to enhance factor reliability (*59*). A 0.5 threshold ensures that all variables in the factor will exhibit at least a moderate relationship with the factor. It also indicates that the factor explains at least 25% of the variance in each observed variable. Factorability of the samples was assessed before the analysis by confirming that all variables correlate significantly to at least one other variable (r ≥ 0.3), by ensuring sufficient levels of sampling adequacy, and by observing that the found correlation matrix is not the identity matrix (a significant result for the Bartlett’s test of sphericity).

### Clustering Analysis

K-means clustering analysis is applied to identify factors leading to increased service adoption among different markets of Bixi users and non-Bixi users. This technique aims to minimize differences within groups while maximizing differences among them. It is based on an iterative centroid method algorithm. Cluster centroids are based on the mean values of the responses for the variables being assessed and are redefined every time a new observation is grouped (*56*). The variables used in the clustering procedure are the factor scores identified in the previous step. The number of clusters was defined based on cluster characteristics, their relevance and transferability to transport policy, previous studies, and common sense and intuition. Complementarily, a silhouette analysis was used to help identify the optimal number of clusters based on the separating distance between them. To evaluate the consistency of the cluster solutions, the analysis was conducted three times while randomly omitting 10% of the observations. Each cluster was characterized based on sociodemographic variables and cycling behavior.

## Results

### Exploratory Factor Analysis

Similar variables were chosen for both users and non-users to generate the factors detailed in Tables 1 and 2, respectively. Specifically, variables related to potential improvements to the service, and if said changes would lead to an increase in their usage of Bixi, or its adoption altogether. The generated factors presented similar patterns for both users and non-users. Both subgroups share the identification of four of the five identified factors: bike/station availability, bicycle characteristics, mobile technology access, and child-oriented features. The fifth factor for each group presents a slight difference between subgroups, where system convenience was identified for non-users, and a more specific factor—membership flexibility—was identified for Bixi users. Each subsample’s factors were used to classify groups that differed in what they desired more out of the service and what challenges they faced in their use, or lack thereof.

**Table 1.** Factor Loadings for the Sample of Bixi Users

Variable (agreement with statement)	Loading	Cronbach’s alpha
Bike/station availability		
I would ride Bixi more often if more docks were added near my home or destination.	0.87	0.78
I would ride Bixi more often if more Bixi bicycles were added near my home or destination.	0.84	
I consider the following to be a challenge: There are no or not enough stations near my home.	0.53	
Child-oriented features		
I would ride Bixi more often if Bixis with child’s seats were introduced.	0.9	0.80
I consider the following to be a challenge: there are no Bixis with child’s seats.	0.74	
Mobile technology access		
I consider the following to be a challenge: I do not have access to internet on my phone.	0.86	0.68
I consider the following to be a challenge: I do not have access to a smart phone.	0.76	
Bicycle characteristics		
I consider the following to be a challenge: weight of the Bixis.	0.84	0.79
I would ride Bixi more often if lighter Bixis were introduced.	0.64	
I consider the following to be a challenge: size of the Bixis.	0.46	
Membership flexibility		
I would ride Bixi more often if a weekly pass was offered.	0.82	0.71
I would ride Bixi more often if a daily pass was offered.	0.66	

*Note*: Variance explained (58.3%); Kaiser-Meyer-Olkin [KMO] (0.580); Bartlett’s test of sphericity (χ^2^ = 1,859.84, degrees of freedom = 66, p-value = 0).

**Table 2.** Factor Loadings for the Sample of Non-Bixi Users

Variable (agreement with statement)	Loading	Cronbach’s alpha
System convenience		
I would ride Bixi if a daily pass was offered.	0.70	0.84
I would ride Bixi if a weekly pass was offered.	0.67	
I would ride Bixi if the $100 credit hold on single rides was removed.	0.67	
I would ride Bixi if a membership discount was introduced for low-income people.	0.65	
I would ride Bixi if the 45-min time limit was extended without additional costs.	0.64	
I would ride Bixi if alternative payment methods were introduced.	0.6	
I would ride Bixi if a free 15-min ride was offered to transit-pass holders.	0.52	
Bicycle characteristics		
I don’t ride Bixi because Bixis are too heavy.	0.85	0.82
I don’t ride Bixi because Bixis are too big.	0.84	
Bike/station availability		
I would ride Bixi if more stations were added near my home or destination.	0.92	0.91
I would ride Bixi if more Bixi bicycles were added near my home or destination.	0.8	
Mobile technology access		
I don’t ride Bixi because I don’t have access to a smartphone.	0.63	0.58
I don’t ride Bixi because I don’t have access to internet on my phone.	0.59	
Child-oriented features		
I would ride Bixi if Bixis with child’s seats were introduced.	0.6	0.44
I don’t ride Bixi because I can’t bring my children with me.	0.51	

*Note*: Variance explained (52.4%); Kaiser-Meyer-Olkin [KMO] (0.760); Bartlett’s test of sphericity (χ^2^ = 4,051.97, degrees of freedom = 105, p-value = 0).

### Cluster Analysis

Based on the factors reported in the previous step, k-means cluster analysis was performed for Bixi users and non-users independently. For both subgroups, a solution of four clusters was found to provide the best description of the market.

#### Bixi Users

**Figure 2.**
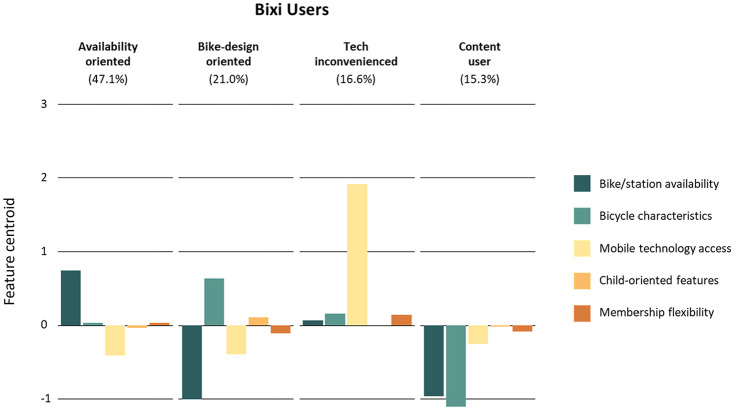
Bixi user cluster groups.

The four identified clusters among Bixi users are: availability-oriented riders, bike-design-oriented riders, tech-inconvenienced riders, and content (happy/satisfied) riders (Figure 2). Table 3 reports on the sociodemographic characteristics of each cluster, residential BikeScore levels, and average weekly cycling trips by bicycle type.

**Table 3.** Descriptive Statistics for Bixi-User Clusters

Variable	Availability oriented (47.1%)	Bike-design oriented (21.0%)	Tech inconvenienced (16.6%)	Content user (15.3%)	All Bixi users
*N*	264	118	93	86	561
Gender (%)					
Man	59.9	55.1	49.5	70.9	58.8
Woman	38.3	40.7	43.0	25.6	36.9
Other	1.9	4.2	7.5	3.5	4.3
Yearly household income (%)					
$60 k or less	15.8	13.6	25.0	16.5	17.7
$60–150 k	51.5	47.6	54.8	39.2	48.2
$150 k or more	32.8	38.8	20.2	44.3	34.0
Age (%)					
18–29 years	11.7	8.5	19.4	5.8	11.4
30–49 years	54.9	67.0	59.1	61.6	59.2
50–64 years	29.2	22.0	16.1	24.4	24.8
65+ years	4.2	2.5	5.4	8.1	4.6
Distance to Bixi station (%)					
Within 250 m	74.6	89.0	81.7	76.7	79.1
Home location BikeScore (%)					
0–49	1.5	0.0	2.2	1.2	1.3
50–69	7.6	2.5	7.5	4.7	6.1
70–89	30.7	22.0	26.9	27.9	27.8
90–100	60.2	75.4	63.4	66.3	64.9
Weekly cycling frequency—mean (SD)					
Personal bicycle	4.67 (4.76)	5.31 (5.33)	5.86 (5.83)	6.29 (5.76)	5.29 (5.28)
Traditional Bixi	2.25 (3.41)	1.51 (3.23)	1.95 (3.07)	2.28 (3.91)	2.08 (3.40)
Electric Bixi	0.58 (1.53)	0.23 (0.90)	0.32 (0.90)	0.28 (0.89)	0.42 (1.24)

Availability-oriented riders’ (47.1% of Bixi users) biggest distinction from other user groups is their likelihood to cite the availability of both stations and bikes as the limiting factor to their bike-sharing use. When compared with the full sample, this group has the lowest share of people living close to a Bixi station, as well as the lowest average BikeScore rating of all Bixi-user clusters. Conversely, this group is also found to have the highest proportion of Bixi use in their weekly trips, with approximately 38% of their cycling trips completed using Bixi.

Bike-design-oriented riders (21% of Bixi users) distinguished themselves by commonly associating the physical attributes of Bixi bicycles (i.e., bike weight and size) as a challenge to using the service. However, contrary to the challenges faced by availability-oriented riders, bike-design-oriented riders typically did not agree that an increase in the availability of bikes/stations in their area would increase their usage of the service. An explanation is that respondents in this cluster already live near Bixi stations and in areas with relatively high BikeScore ratings.

Tech-inconvenienced riders (16.6% of Bixi users) uniquely associated their current Bixi use with challenges brought on by the mobile technology required to use the service (i.e., access to a phone, data plan, or both). This might be explained by the demographics of this group. They, on average, have the lowest income and are the youngest compared with other user groups; therefore, they are potentially in a position where getting access to a smartphone or data plan is a challenge.

Content riders (15.3% of Bixi users) report mostly not facing challenges while using Bixi. They also do not indicate any service improvements that would increase their Bixi usage. This group distinguishes itself from others mostly in sociodemographic characteristics, with higher shares of high-income older-adult male riders. Moreover, they tend to live close to Bixi stations and in areas with relatively high BikeScores. They have the highest frequency of cycling trips and have the second highest use of Bixi when compared with other user groups.

### Non-Bixi Users

**Figure 3.**
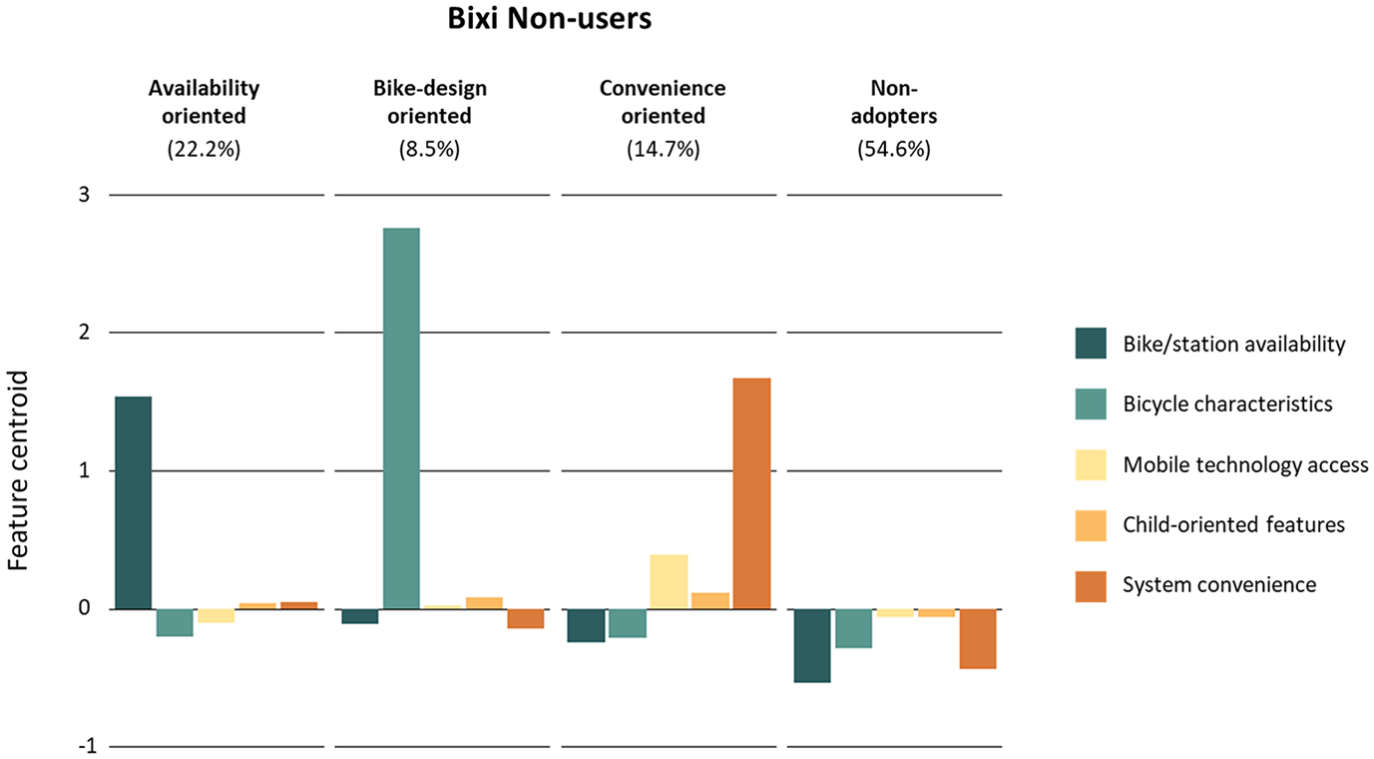
Non-Bixi-user cluster groups.

The four groups resulted from the k-means clustering analysis for the non-users were: availability-oriented riders, bike-design-oriented riders, convenience-oriented riders, and non-adopters (Figure 3). Table 4 reports on the sociodemographic characteristics of each cluster, their residential BikeScore levels, and their average weekly cycling trips by bicycle type.

**Table 4.** Descriptive Statistics for Non-Bixi-User Clusters

Variable	Availability oriented (22.2%)	Bike-design oriented (8.5%)	Convenience oriented (14.7%)	Non-adopters (54.6%)	All Bixi non-users
*N*	169	65	112	417	763
Gender (%)					
Man	56.8	50.8	53.6	62.1	44.7
Woman	39.6	41.5	36.6	34.5	30.5
Other	3.6	7.7	9.8	3.4	4.9
Yearly household income (%)					
$60 k or less	18.2	25.9	36.5	12.1	23.0
$60–150 k	51.3	48.3	44.8	54.2	49.7
$150 k or more	30.5	25.9	18.8	33.7	27.3
Age (%)					
18–29 years	2.4	6.2	6.3	2.9	3.5
30–49 years	49.1	69.2	38.4	38.9	43.6
50–64 years	33.1	24.6	42.0	37.4	36.0
65+ years	15.4	0.0	13.4	20.9	16.8
Distance to Bixi station (%)					
Within 250 m	45.0	70.8	71.4	66.4	62.8
Home location BikeScore (%)					
0–49	4.1	1.5	0.9	1.2	1.8
50–69	22.5	13.8	14.3	11.8	14.7
70–89	39.1	26.2	26.8	42.2	37.9
90–100	34.3	58.5	58.0	44.8	45.6
Weekly cycling frequency—mean (SD)					
Personal bicycle	6.46 (5.23)	5.82 (5.10)	7.87 (6.21)	6.08 (5.06)	6.32 (5.30)

Availability-oriented cyclists (22.2% of non-users) attribute not using Bixi to the non-existent or limited availability of stations around their home and desired destinations. Like the equivalent Bixi user segment, availability-oriented cyclists have the lowest share of people living close to a Bixi station and the lowest average BikeScore of any cluster. Only 45% of its members live within 250 m of a Bixi station. This is also reflected in the mean number of weekly trips (5.8), which is, again, the lowest of any cluster.

Bike-design-oriented cyclists (8.5% of non-users) associate not using Bixi services with the physical characteristics of the bikes available in the system, citing them as too heavy, too big, or both. They were also the group with the largest share of women and were found to be substantially younger than the rest of the non-user clusters. They exhibit a relatively high number of average weekly trips and tend to live in areas with a high BikeScore, suggesting that they prefer the features of their personal bikes to Bixi bicycles.

Convenience-oriented cyclists (14.7% of non-users) cite membership- and payment-flexibility-related reasons as the main reason for not using Bixi services. In addition, access to the mobile technology required to access the Bixi service is another factor for their lack of engagement. Respondents in this group have the highest share of people living near a Bixi station among non-users. They also complete the highest number of weekly cycling trips compared with other non-user groups.

For non-adopters (54.6% of non-users), no improvements made to Bixi services would get them to adopt the service. This group is demographically similar to the content Bixi user segment. They are substantially more male dominated, are older, and typically have higher income levels.

## Discussion

The results in this study provide several insights into the barriers and challenges perceived by users and non-users of a bicycle sharing system, highlighting key areas for improvement. While users, having direct experience with the system, may be more aware of certain challenges than non-users, the five factors and four profiles identified for each subsample were found to be fairly analogous between users and non-users. This indicates that most potential improvements to the bike-sharing system would, in general, have positive impacts both in increasing usage of current users, as well as attracting new riders. However, key differences are found between the perceptions of users and non-users, which give a deeper perspective into each group’s unique needs and attitudes toward bike-sharing usage and potential adoption. The insights found through these results can have multiple implications for public policy developers aiming to enhance the reach of these programs and promote positive equity outcomes through their effective implementation.

**Figure 4.**
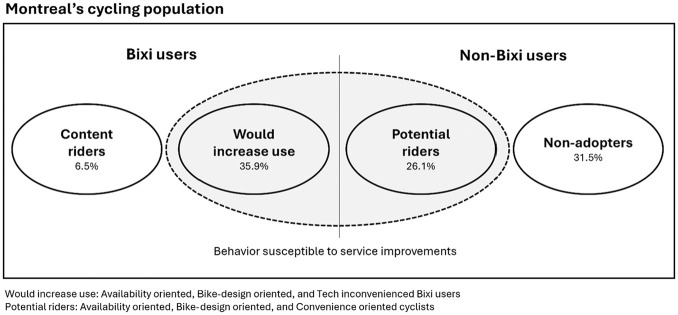
Overview of the Bixi market.

Figure 4 provides an overview of the bike-sharing market in Montréal. While content Bixi riders and non-adopters are unlikely to change their behavior, other groups identified in our analysis have the potential to either increase or adopt bike-sharing once service improvements respond to their concerns.

The two profiles most concerned with the availability of Bixi services for both user and non-user groups, are characterized by the lowest spatial availability of Bixi stations when compared with other segments. This results in bike-sharing becoming an inconvenient or unfeasible option for these groups. Availability-oriented riders make up the largest group of Bixi users. Among non-users, they form the second largest cluster, after non-adopters, making them the largest group of potential new users. This means that the spatial expansion of service to reach the participants in these groups would likely have the greatest impact in Bixi usage overall. Previous literature indicates that the increase in availability should be accomplished through adding more stations, as it has been found to have a greater impact on bike-sharing usage than increasing the number of bikes at a given station (*24*). Expanding the number of stations also poses benefits to decreasing the vulnerability of the system at large (*29*).

Membership flexibility—a factor measuring if the addition of alternative membership models would increase the usage of existing Bixi users—was not found to be a significant challenge for any of the identified user groups. However, among non-users, the combination of membership flexibility components and fare/cost-related challenges are the most significant barriers for convenience-oriented cyclists to adopt bike-sharing services. Respondents in this group are characterized by generally lower income than other non-user clusters. On average, they live in areas with good spatial access to Bixi and higher BikeScore, and make substantially more cycling trips. This means that, although Bixi is accessible to them, cost- and membership-related barriers stop them from potentially becoming frequent Bixi users. In this sense, cost- and membership-related measures could have the largest impacts in promoting Bixi ridership in lower-income groups currently not using the system.

The group of Bixi users whose main concerns are related to the mobile technology required to use the service (i.e., access to a phone/mobile data) tends to have higher shares of younger low-income members. This concern is shared by the convenience-oriented non-user group. Because these two groups have the lowest incomes within their respective subsamples, measures addressing the inconvenience caused by the requirement of a smartphone and mobile data can have positive equity impacts on income. The introduction of alternative payment methods and Wi-Fi access at stations could potentially increase use/adoption of Bixi services among these lower-income groups.

The challenges related to the physical attributes of Bixi bikes, such as weight and size, were more predominant concerns for riders who already have good Bixi coverage. This can be concluded seeing as they share a disinterest in questions associated with increasing the availability of bikes and stations around them. They also tend to live in areas with high BikeScore ratings. This suggests that implementing changes that would accommodate the challenges associated with the bike design are secondary to other more pertinent measures, such as the reach and availability of the service. Furthermore, changing and improving the design of the bicycles themselves would be harder to implement and adequately apply in the near term as it would entail larger financial investments.

A relevant result of this work lies in the identification of groups for which no improvements can potentially change their use (or non-use) of the Bixi system. Non-users, the largest identified group, are not susceptible to improvements to Bixi, removing them from the pool of future potential bicycle-sharing users. Similarly, a portion of existing Bixi users indicate no major challenges in their use of the service, and therefore no potential improvements would lead them to increase their usage. Notably, both groups share similar demographic features, with higher household income and a higher share of men than other groups. This finding is corroborated by the literature, as bike-sharing members tend to be younger (*1*, *5*).

## Conclusions

Bike-sharing services have been gaining popularity serving as a potential innovative solution to increase shares of sustainable trips and physical activity. Even though several studies have explored the determinants of bike-sharing ridership, few studies have tried to understand the heterogeneous needs within this market. This paper addresses this gap by exploring factors potentially limiting cyclists use of a public bike-sharing service (Bixi) in Montréal, Canada. Samples of both users and non-users are examined through a combination of factor and cluster analysis. A solution of four clusters was found to provide the best description of the market for each group. Each cluster represents a potential direction to increase bike-sharing usage/adoption based on the specific needs and perceptions of its members.

The findings of this study highlight several practical recommendations for planners and policymakers to enhance bike-sharing systems. Increasing availability of bikes and stations, particularly in underserved areas, may have the largest impact in increasing ridership of both current users and potential new users. In this sense, operators should examine its bike flows to determine the most optimal areas to expand its coverage. When focusing on improving conditions for equity-seeking groups, providing alternatives to requiring a smartphone and mobile data can have the largest impact on increasing use by low-income current users. This can be addressed by providing cash or card payment options, as well as by providing Wi-Fi at stations. Meanwhile, improving system convenience (i.e., offering alternative pass structures) and reducing costs can have the largest impact in bringing low-income new users. Offering flexible and affordable membership models, such as subsidized passes or pay-as-you-go options, can help reduce cost barriers and encourage adoption among low-income non-users. These targeted measures can make bike-sharing more accessible, equitable, and appealing to a broader population.

Potential limitations can be identified for this study. First, using a cross-sectional survey limits our ability to establish causal relationships between the identified factors and bike-sharing adoption. Second, while the sample size of 1,324 cyclists used in this work provides valuable insights into system-wide trends, this approach may overlook smaller groups that could experience different barriers to adoption or usage. Lastly, while cyclist profiles are derived through factor and k-means cluster analyses, these methods may not capture the full complexity of individual behaviors. Future research could consider a more targeted approach, exploring the needs of these smaller segments, and validating the broader trends identified in this study.

In this line, future research can build on this study by applying qualitative methods, such as thematic analysis and in-depth interviews, to further explore the challenges experienced by users and non-users alike. Future research can also explore segments of the population who do not cycle to understand their unique needs and concerns about adopting bike-sharing systems. Future studies could also examine the challenges posed by mobile technology, particularly how issues related to app usability differ across age groups and income levels. Additionally, research could explore how users’ perceptions of bike-sharing systems evolve after they begin using the service. This would require panel data to track changes in attitudes over time. Understanding how purchasing a personal bike or e-bike affects the usage patterns of bike-sharing systems could provide insights into the factors that influence the transition between shared and private cycling. Another promising avenue is exploring how cyclists balance concerns about the ride experience itself versus utilitarian considerations, such as using the system as a means to reach a destination. Finally, investigating how safety concerns vary across different demographic groups could provide insights into how to make bike-sharing systems more accessible and appealing to a wider range of potential users.
